# Activities of Clinical Expertise and Research in a Rare Disease Referral Centre: A Place for Telemedicine beyond the COVID-19 Pandemic?

**DOI:** 10.3390/healthcare11172447

**Published:** 2023-08-31

**Authors:** Quentin Ducrocq, Laurence Guédon-Moreau, David Launay, Louis Terriou, Sandrine Morell-Dubois, Hélène Maillard, Guillaume Lefèvre, Vincent Sobanski, Marc Lambert, Cécile Yelnik, Meryem-Maud Farhat, Maria José Garcia Fernandez, Eric Hachulla, Sébastien Sanges

**Affiliations:** 1 CHU Lille, Service de Médecine Interne et Immunologie Clinique, Centre de Référence des Maladies Auto-Immunes Systémiques Rares du Nord et Nord-Ouest de France (CeRAINO), F-59000 Lille, France; quentin.ducrocq@chu-lille.fr (Q.D.); david.launay@univ-lille.fr (D.L.); louis.terriou@chu-lille.fr (L.T.); sandrine.dubois@chu-lille.fr (S.M.-D.); helene.maillard@chu-lille.fr (H.M.); vincent.sobanski@chu-lille.fr (V.S.); marc.lambert@chu-lille.fr (M.L.); cecile.yelnik@chu-lille.fr (C.Y.); meryem.farhat@chu-lille.fr (M.-M.F.); eric.hachulla@chu-lille.fr (E.H.); 2 Université de Lille, Faculté de Médecine et CHU de Lille, Clinique de Cardiologie et Maladies Vasculaires, F-59000 Lille, France; laurence.guedon@chu-lille.fr; 3 Univ. Lille, U1286—INFINITE—Institute for Translational Research in Inflammation, F-59000 Lille, France; guillaume.lefevre@chu-lille.fr; 4 Inserm, F-59000 Lille, France; 5 Health Care Provider of the European Reference Network on Rare Connective Tissue and Musculoskeletal Diseases Network (ReCONNET), F-59000 Lille, France; 6 CHU Lille, Laboratoire d’Immunologie, F-59000 Lille, France; 7 CHU Lille, Département de Médecine Polyvalente Post-Urgences, F-59000 Lille, France; 8 Univ. Lille, U1167—RIDAGE—Risk Factors and Molecular Determinants of Aging-Related Diseases, F-59000 Lille, France; 9 Unité Matériaux et Transformations (UMET) UMR CNRS 8207, Université Lille 1, F-59655 Villeneuve d’Ascq, France; maria-jose.garcia-fernandez@univ-lille.fr; 10 Inserm, CHU Lille, U1008—Controlled Drug Delivery System and Biomaterials, University Lille, F-59000 Lille, France

**Keywords:** telemedicine, telehealth, teleconsultation, tele-expertise, tele-trial, rare diseases, auto-immune diseases, referral centre, clinical research, clinical trial

## Abstract

Introduction: Rare disease referral centres are entrusted with missions of clinical expertise and research, two activities that have to contend with numerous obstacles. Providing specialist opinions is time-consuming, uncompensated and limited by difficulties in exchanging medical data. Clinical research is constrained by the need for frequent research protocol visits. Our objective was to determine whether telemedicine (TLM) can overcome these difficulties. Methods: To better characterise the activity of clinical expertise provided by our French centre, each opinion delivered by our team was reported on a standardised form. To investigate our clinical research activity, investigators and patients were asked to complete a questionnaire on the acceptability of research protocol teleconsultations. Results: Regarding clinical expertise, our team delivered 120 opinions per week (representing a total of 21 h), of which 29% were delivered to patients and 69% to medical practitioners. If these were delivered using TLM, it would represent a potential weekly income of EUR 500 (tele-expertise) and EUR 775 (teleconsultations). Regarding the research activity, 70% of investigators considered the frequency of visits to be a limiting factor for patient inclusions; nearly half of the patients surveyed would be in favour of having teleconsultations in place of (40%) or in addition to (56%) in-person visits. Conclusion: Whereas TLM has become widely used as a back-up procedure to in-person consultations during the COVID-19 pandemic, the solutions it provides to the problems encountered in performing expertise and research activities have made it a new conventional follow-up modality for patients with rare diseases.

## 1. Introduction

The European Union considers a disease to be rare when it affects less than 1 in 2000 persons [1]. Although each of an estimated 6–8000 rare diseases is individually uncommon, these conditions collectively affect between 27 and 36 million Europeans, making them a public health issue [1,2]. In France, they affect nearly 3 million people [3]. The causes of RDs are often elusive, with the most common etiologies being genetic (40–80%) and immune-mediated [4]. Rare diseases are often associated with poor prognosis [2,4] due to lack of awareness among physicians leading to difficulties in diagnosis (the so-called “diagnostic odyssey”) and few available therapeutic options. To try and deal with these issues, the management of patients with rare disease is usually performed in dedicated expert referral centres.

Rare disease referral centres perform a dual mission: on the one hand, a diagnostic and therapeutic mission, making their expertise available to their patients and to other medical practitioners, and, on the other hand, a clinical research mission, with the ultimate aim of enabling their patients to benefit from the latest available advances in treatment.

Currently, however, these activities are often performed in a sub-optimal manner due to various obstacles. Regarding provision of expertise, this activity is now partly conducted via phone or email, due to the increasing strain on consulting and day-patient clinics and the need for an immediate response to problems encountered by colleagues and patients. However, these communication techniques suffer from inherent weaknesses: limited means of sharing medical data (videoconferencing, photographs, biological samples, imaging), difficulty of ensuring secure data transmission (under French law, fax and post remain the only means of respecting the confidentiality of medical data, given the absence of any generalised health data messaging system in France), lack of traceability of the opinions delivered and lack of visibility and remuneration for an activity that is increasingly time-consuming. Regarding research activity, participation in a clinical trial often means that patients are required to make more frequent follow-up visits to the physician-investigators, which may act as a brake on inclusions and also lead to premature withdrawals from the study. Consequently, to fulfil these two missions, there appears to be a need for an optimised, remote technique.

Telemedicine (TLM) is a form of remote medical practice that makes use of information and communication technologies [5]. It enables health professionals, at least one of whom must be a physician, to communicate with one another or with a patient. The TLM acts officially recognised in France include teleconsultation (TLC), which connects a patient with his/her doctor, and tele-expertise (TLE), which connects a medical practitioner requesting an opinion with the physician responsible for delivering it [6]. After developing gradually over the past few years, in particular to address difficulties in accessing care in so-called medical deserts and due to the growth in the follow-up of chronic diseases, the use of TLM has recently spread massively and rapidly to meet public health requirements during the COVID-19 pandemic [7].

Rare disease referral centres are no exception and have sometimes had to resort to TLCs as an alternative to in-person consultations, especially in the case of patients at risk of developing a severe form of COVID-19. Nevertheless, in view of the obstacles these centres have encountered in their expertise and clinical research activities, one may ask whether TLM could play an integral and long-term role, rather than merely providing an inferior, back-up solution to in-person consultations in a crisis situation.

Our objective was therefore to determine whether TLM could help to resolve the difficulties encountered in the expertise and clinical research activities of a rare disease referral centre. To this end, two surveys were conducted in parallel in our Internal Medicine and Clinical Immunology Department, a French referral centre for rare systemic diseases: firstly, we aimed to better quantify and characterise our expertise activity; secondly, we evaluated the acceptability to physician-investigators and patients of using follow-up TLCs in the context of clinical research protocols.

## 2. Methods

The study was performed among physicians and patients from the Internal Medicine and Clinical Immunology Department (hereafter, “the Department”) of Lille University Hospital, France. It has been labelled a French referral centre for rare systemic auto-immune and auto-inflammatory diseases since 2007 (*Centre de Référence des Maladies Auto-Immunes Systémiques Rares du Nord et Nord-Ouest*, CeRAINO) and follows a cohort of approximately 3000 patients with such conditions. All surveys were administered in French and their English translations are provided in Supplemental Tables S1–S4.

### 2.1. Survey of Expertise Activity

#### 2.1.1. Census of Opinions Given

The survey was conducted among the 11 physicians of the Department (SS, DL, LT, SMD, HM, GL, VS, ML, CY, MMF and EH) during a six-week period (1 October 2018 to 11 November 2018), comprising a standard three-week period (1 to 21 October 2018) and an off-peak three-week period that coincided with school and public holidays (22 October to 11 November 2018). During the period of the survey, the physicians were asked to complete an anonymous online form each time they gave a specialist opinion via phone or email. To try to ensure that data collection was as exhaustive as possible, an icon with a shortcut to the form’s webpage was installed on the physicians’ smartphone home screens, and the webpage was also included on the start pages on their desktop browser’s homepage.

The form comprised 11 questions regarding the characteristics of the expert opinions given (Supplementary Table S1): date, contact method (email or phone), status of the request, whether a bedside visit was required, conclusion (hospitalisation, consultation, reorientation), total time devoted. Each form took about 1 min to complete.

#### 2.1.2. Quality of the Census

At the end of November 2018, approximately two weeks after the end of the survey period, the physicians were invited to provide feedback by anonymously answering an online questionnaire sent via email. The questionnaire consisted of 16 questions and was designed to evaluate the degree of exhaustiveness of the declared opinions, the level of precision of the declared durations and the daily inconvenience of reporting opinions (Supplementary Table S2). The questionnaire took less than 5 min to complete.

#### 2.1.3. Medico-Economic Evaluation of the Expert Opinion Activity if Performed as TLM Acts

Under French regulations [8,9], expert opinions delivered to patients and to physicians can qualify as TLC and TLE acts, respectively, if performed using a secured communication platform (that must include video-transmission in the case of TLCs). TLC and TLE acts can be compensated at a unit cost of EUR 28 and EUR 20, respectively.

### 2.2. Survey of the Clinical Research Activity

Two questionnaires were prepared, one to obtain the views of physician-investigators and the other to obtain the views of patients included in clinical research protocols.

#### 2.2.1. Physician Survey

The physician survey was conducted among the 10 physicians of the Department who had already acted as investigators in a clinical research study and conducted protocol visits (SS, DL, LT, SMD, HM, GL, VS, ML, CY and EH). The physicians were invited to anonymously complete a questionnaire containing 10 questions on their previous experience in clinical research, their knowledge of TLM and their views on the potential interest of study protocol follow-up TLCs (Supplementary Table S3). The questionnaire took less than 5 min to complete.

#### 2.2.2. Patient Survey

The patient survey was conducted among patients with rare auto-immune or auto-inflammatory diseases who had been included in a clinical research study for at least 1 month and had agreed to complete a questionnaire on their views. The survey took place over a period of 5 months (March to July 2019), which enabled us to include a representative panel of 50 unselected consecutive patients, each of whom completed the self-administered questionnaire once. The questionnaire, completed anonymously, consisted of nine questions relating to the frequency of protocol visits, patients’ previous experience of TLM and their views on the potential interest of study protocol follow-up TLCs (Supplementary Table S4). The questionnaire took less than 5 min to complete.

## 3. Results

### 3.1. Characterisation of the Expertise Activity of Our Department

#### 3.1.1. Survey of the Opinions Delivered

**Figure 1:**
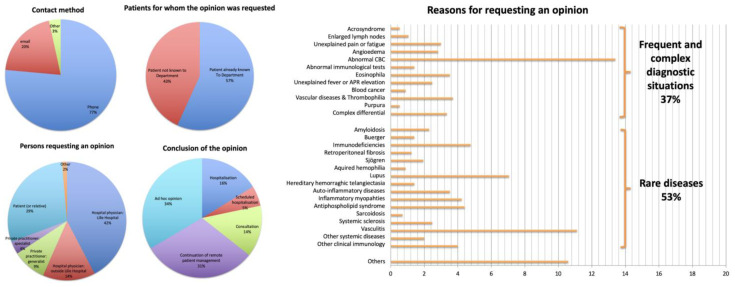
Results of the survey regarding specialist opinions delivered during the study period. CBC: complete blood count; APR: acute phase reactants.

In the first step, we aimed to characterise the expertise activity of our Department by carrying out an exhaustive census of the specialist opinions delivered during the study period (Figure 1). A total of 567 opinions were registered, including 355 for the first 3 weeks (a period outside the physicians’ annual leave), thus representing nearly 120 opinions per week in a standard period. Overall, this activity represented a total of 105 h, of which 63 h were in the first three weeks, i.e., 21 h per week during this period. Opinions were essentially delivered via phone (75% of cases) and email (20%). In 96% of cases, the physician receiving the request did not make a bedside visit; this percentage fell to 91% of cases for opinions delivered to persons within our hospital. However, bedside visits accounted for a considerable amount of time (15 h, or 29% of the overall time devoted to opinions).

The opinions concerned patients already followed up in our Department in 57% of cases. The reasons for seeking an opinion were very varied, but could be grouped into two situations: diagnostic or therapeutic management of rare diseases (e.g., systemic sclerosis, vasculitis, systemic lupus erythematosus and complications of immunosuppression) in 53% of cases, and management of frequent complex diagnostic situations (e.g., unexplained asthenia/pain, chronic fever of unknown origin, non-inflammatory vascular disease/thrombophilia, angio-œdema) in 37% of cases.

The main categories of persons requesting an opinion were hospital physicians at Lille University Hospital (42%) or another hospital (14%), the patient or a relative (29%) and practitioners in private practice, either generalists (9%) or specialists (4%). Physicians requesting opinions worked in medical specialty departments (38%), critical care medicine (22%), surgical and obstetrics–gynaecology departments (16%), general family medicine (13%) and internal medicine departments of other centres (7%). The hospital physicians from other centres worked in our region (77%), in the Amiens-Caen-Rouen sub-region (6%), elsewhere in France (14%) or in another country (4%).

The opinion could be delivered on an ad hoc basis (34%), require the patient’s management to be continued remotely (31%), or lead to an in-person referral in our Department (35%, of which 14% were consultations, 21% were in-patient hospitalisations and 5% were day-patient hospitalisations).

#### 3.1.2. Quality of the Census Carried Out

In a second step, we aimed to assess the quality of the data collected during the census period with the help of a feedback questionnaire. The questionnaire was completed by all of the physicians who took part in the census.

In general, the participants considered they had been “very” (64%) or even “entirely” (27%) exhaustive in their declarations: nearly half of the respondents considered they had forgotten fewer than two opinions per week (45% of respondents in regular weeks and 43% in weeks on call). The reported level of exhaustiveness was higher for opinions given via phone (91%) or involving a bedside visit (100%) than for those given by email (73%). There was no apparent disparity in the reported level of exhaustiveness of declarations between the different categories of requesting persons.

The declared duration of each opinion was rated as “rather” (64%) or “very” (27%) precise by respondents. The task of declaring opinions was generally considered “very little” or “not at all” time-consuming (82%), and “very little” or “not at all” tedious (82%). However, the census forms were completed at the time the opinion was given by only 45% of respondents.

Taken together, the feedback survey data suggest that the census was reliable, exhaustive and representative.

#### 3.1.3. Medico-Economic Evaluation of the Expert Opinion Activity if Performed as TLM Acts

As most of our expert opinions were provided via phone or email, they cannot readily qualify as TLM acts under French law [8,9]. Interestingly however, if the opinions delivered to patients were performed using a secured video-transmission platform, they would meet the regulatory requirements for TLCs and, as such, they would allow for compensation of EUR 28/TLC, which represents approximately EUR 775/week according to our census data. Similarly, if the opinions provided to physicians outside of our hospital used a secured communication channel, they could be considered as TLE acts and be compensated at an amount of EUR 20/act, which represents approximately EUR 500/week according to our census data.

### 3.2. Acceptability of Protocol Follow-Up TLCs to Physician-Investigators and to Patients Included in Clinical Research Protocols

We then sought to determine the acceptability of using TLCs for clinical research protocol follow-ups. To this end we used a questionnaire to elicit the views of physician-investigators and included patients.

#### 3.2.1. Physician Survey (Figure 2)

**Figure 2:**
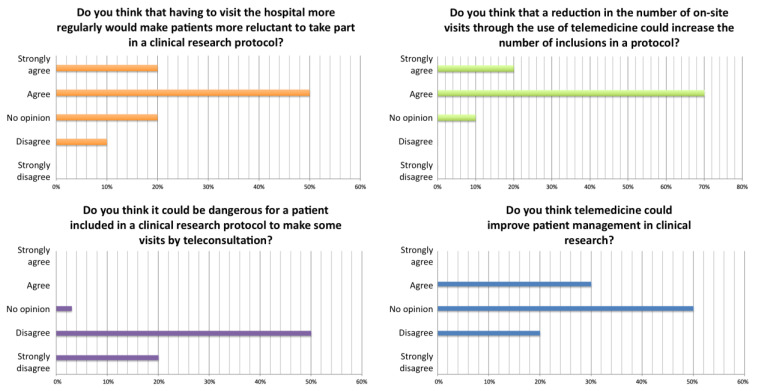
Results of the survey regarding physician-investigators’ opinions on telemedicine and clinical research.

All of the physicians surveyed (100%) had already heard of TLM, had acted as an investigator in a clinical research study, had included patients in a study and had conducted or participated in a protocol visit (Figure 2).

Most of the physicians (70%) considered that having to visit the hospital more frequently within the framework of a study protocol was a potential disincentive for a patient to participate, and that reducing the number of in-person visits through the use of TLCs could encourage inclusions. Most of the physicians considered that recourse to protocol follow-up TLCs would be more convenient for patients (50%) and pose little risk (70%), but would also be more time-consuming for themselves than in-person consultations (60%). However, only 30% considered that the use of TLCs would help to improve patient management.

#### 3.2.2. Patient Survey (Figure 3)

**Figure 3:**
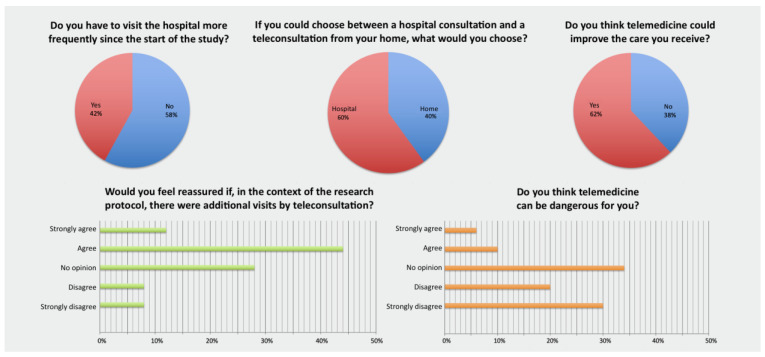
Results of the survey regarding patients’ opinions on telemedicine and clinical research.

A majority of the patients (58%) did not have the impression they needed to visit the hospital more frequently since their inclusion in the study, nor did the majority of patients (76%) experience organisational difficulties in keeping their appointments in the Department (Figure 3).

Most patients (74%) had already heard of TLM. Only 40% of them would prefer a follow-up TLC to an in-person consultation at the hospital if they could choose. However, 56% of patients said they were “very” or “completely” reassured if protocol TLCs were in addition to scheduled, in-person visits, and 62% considered that TLM could help to improve their overall care. Most of them thought that the use of TLM for certain protocol visits would be more convenient (75%), less time-consuming (75%) and pose little risk (50%).

## 4. Discussion

Rare disease referral centres are tasked with providing the best available care to their patients. This includes two complementary and equally important missions. First, they ensure that patients receive an optimal evaluation in terms of diagnosis and therapeutic options. Quite often, this consists mostly of providing clinical counseling to other practitioners unfamiliar with such conditions. Recent studies show that rare disease awareness is insufficient among senior physicians and medical students, partly due to inadequate academic training [10,11,12]. Consequently, delivering expert opinions is an essential part of the daily life of rare disease centres, one that is highly time-consuming, insufficiently visible and acknowledged and often inadequately compensated. Second, they serve as investigating centres for clinical research protocols, in order to advance knowledge in the field of rare diseases and to provide their patients with innovative therapies. As the number of such centres is limited, patients may have to travel long distances to participate in a clinical trial. Our study suggests that these issues could benefit from an optimized remote option, such as TLM.

Our study draws strength from its setting in a high-profile French rare disease centre, which allowed us to collect representative data both in terms of clinical expertise and research. It also has limitations. First, the data collected are mostly declarative and descriptive. However, careful attention was given to ensure their exhaustivity and representativeness, to make results as accurate as possible. Second, detailed patient data (and especially rare disease diagnosis) were not collected in the research survey. However, since our Department mostly participates in protocols related to connective tissue diseases, it is reasonable to assume that the patients included in our panel were diagnosed with such conditions. Third, since data suggest that the COVID-19 pandemic had a major impact on rare disease patient management (especially in terms of the use of TLM) [13], it would have been interesting to assess the evolution of our activities of clinical expertise and research during and after lockdown. Unfortunately, this was not possible as our data were collected before the COVID-19 pandemic outbreak. However, as our data demonstrate a potential place for TLM even before the pandemic, we believe that it further reinforces its relevance as one of the various modalities of care available for the management of patients with rare diseases. Finally, our study is relevant in the context of the French healthcare system, and as such, its results may not be easily extrapolated to other settings.

### 4.1. Providing Clinical Expertise Is a Time-Consuming and Uncompensated Activity: Could TLM Be a Solution?

Delivering clinical expertise in complex diagnostic situations and rare diseases is a core activity of referral centres. While most physicians empirically consider that it involves a substantial volume of work, our study provides an objective and quantified evaluation of this activity, demonstrating how time-consuming it actually is. Carrying out this activity involves a number of challenges, in particular the lack of compensation and the difficulty of exchanging components of a patient’s medical file via secure channels. TLM can offer a solution to these problems, as most of the expert opinions delivered to patients or physicians could qualify as TLC or TLE acts under French law, if performed using appropriate channels (notably, secure videoconferencing instead of phone calls for TLCs). A medico-economic assessment based on the findings of our study indicated that this specialist opinion activity could be valued at around EUR 65–70,000 per year, a budget that could finance investment in the equipment (especially IT equipment) and human resources (dedicated physician-time) needed for its deployment.

Other studies have also reported the value of TLM in the expertise activity of rare disease referral centres. Even before the emergence of the COVID-19 pandemic, physicians at the centre in Clermont-Ferrand, France were already reporting on their experience with a TLE regional platform [14], set up to help general practitioners faced with difficult diagnostic situations: the platform enabled patient care to be more precisely directed and it significantly reduced the mean number of hospitalisations. Likewise, the team of the French national reference centre for thrombotic microangiopathies reported having set up a hub to provide ‘pseudo’ TLM via phone and email [15], enabling them to deliver urgent, specialist advice in real time 24/7 and thereby improve the prognosis for patients with these potentially life-threatening diseases. Further afield, an Australian team reported on their experience with TLCs, provided by rheumatologists at the Woolloongabba University Hospital for patients with an inflammatory disease, during an appointment with a trained nurse at their local hospital [16]; this arrangement allowed a considerable reduction in patients’ travel time and most of them expressed overall satisfaction. Furthermore, two systematic reviews of the international literature collated the results of around 20 studies on the use of TLM in the follow-up care of patients with chronic inflammatory rheumatism or connective tissue disease [17,18]: virtually all of them suggested good feasibility of TLC in rheumatology, physician and patient satisfaction with this mode of healthcare and its likely equivalence when compared to conventional care (though with a low level of evidence).

From the start of the COVID-19 crisis, other rare disease centres [19,20,21] quickly had to deploy a means of remote consultation and they found the experience to be very positive, even emphasising that TLC could usefully be included in some patients’ standard follow-up after the pandemic.

There are nevertheless some limitations. In France, the regulations governing TLM acts stipulate the use of a secure tool (including videoconferencing in the case of TLC), which may therefore require logistic reorganisation of the system for requesting and providing expertise (a system often based solely on phone or email), and an investment in equipment (computers, webcams, headphones, microphones) for both doctor and patient. Both these actors need to be trained in using the tools and in the practice of TLC itself, and provisions must be made for reactive technical maintenance. Moreover, the long-term security of this remote modality has yet to be formally ascertained, both in terms of patient management (control of the disease and tolerance to treatments) and in terms of the link between physician and patient (breakdown of the care relationship).

### 4.2. Clinical Trial TLCs: Towards Tele-Trials?

The management of rare and mostly orphan diseases often requires our patients to be included in clinical research protocols. As referral centres generally draw their patients from a very wide area, study inclusions and protocol follow-ups are often limited due to travel and access problems. Quite surprisingly, while a majority of the physician-investigators questioned considered the frequency of in-person visits as a brake on inclusions, most of the patients surveyed did not have the impression that their hospital visits had increased since the start of the study and, as a result, were not in favour of these visits being replaced by a remote modality. This apparent discrepancy may have been due to a selection bias, given that the patients surveyed had already agreed to the study protocol and therefore constituted a cohort undeterred by the constraints involved.

Prior to the COVID-19 pandemic, TLM was used anecdotally in the context of clinical trials (‘teletrials’), particularly in the fields of oncology and dermatology [22,23,24,25,26]. These first experiences already suggested the potential of this follow-up modality: for example, in the context of a study on thromboembolism prevention for cancer patients, the team of Lee et al. offered included patients in-person visits or TLCs [26] and observed no significant difference in levels of distress and discomfort between the two groups. Furthermore, the patients in the TLC group described this follow-up modality as acceptable and promoting equity of access to care.

The advantages associated with having recourse to a remote modality were already being emphasised several years ago [25]. Firstly, TLM enables the participation of patients for whom in-person visits would have been a limiting factor, especially those living in remote, often rural areas far from the investigation centre. Recruiting over a wider area means that a more diverse and therefore more representative sample can be included. Secondly, TLM increases the range of possibilities in terms of modalities of data collection (collection of real-life and real-time data now being possible thanks to connected equipment and smartphone applications) and communication between patients and investigators (e.g., reminders promoting adherence to treatment; reporting of adverse reactions). Lastly, dematerialising all or part of the protocol follow-up can reduce study costs by cutting the number of investigator sites and through the use of automated and centralised data collection.

Some limitations were also identified: logistic constraints specific to the use of TLM (IT equipment and maintenance; ensuring security of data, etc.); lack of a well-defined regulatory framework (in particular with regard to the status of a satellite site where the patient receives the TLC); problems with including computer-illiterate patients, etc.

Since the start of the COVID-19 pandemic, many investigation centres have, in the same way as standard healthcare facilities, been forced to develop remote solutions in order to maintain the study protocol follow-up of their patients, and they are now considering whether to incorporate TLM as a standard modality to be offered during clinical trials [27,28,29]. Various ways of organising this could be envisaged: all or part of the follow-up could be scheduled to take place remotely, depending on the study and the patient’s own preference; or TLC could serve as an ad hoc, compromise alternative to an in-person visit in the event of the patient being otherwise unavailable.

## 5. Conclusions

While relatively rare just a year or two before the COVID-19 health crisis, TLM has, with the advent of the pandemic, become widely used as a temporary back-up procedure to replace in-person consultations. Yet, the concrete and practical solutions that TLM can offer for the everyday problems encountered in the expert advice and clinical research activities of rare disease referral centres will most likely lead to its becoming established as one of the standard follow-up methods we offer our patients.

## Data Availability

The datasets during and/or analysed during the current study are available from the corresponding author on reasonable request.

## Supplementary Materials

The following supporting information can be downloaded at: https://www.mdpi.com/article/10.3390/healthcare11172447/s1, Table S1: Census form for specialist opinions given by physicians of the Internal Medicine—Clinical Immunology Department, CHU Lille, France; Table S2: Feedback questionnaire on specialist opinions given by physicians of the Internal Medicine and Clinical Immunology Department, CHU Lille, France; Table S3: Questionnaire on views about telemedicine and clinical research for physician-investigators at the Internal Medicine and Clinical Immunology Department, CHU Lille, France; Table S4: Questionnaire on views about telemedicine and clinical research for patients included in a clinical research protocol at the Internal Medicine and Clinical Immunology Department, CHU Lille, France.

## References

[1] European Union Committee of Experts on Rare Diseases (EUCERD) (2014). 2014 Report on the State of the Art of Rare Disease Activities in Europe.

[2] Donnart A., Viollet V., Roinet-Tournay M. (2013). [Rare diseases, a public health issue]. Soins Pediatr. Pueric..

[3] Amselem S., Gueguen S., Weinbach J., Clement A., Paul Landais for the RaDiCo Program (2021). RaDiCo, the French National Research Program on Rare Disease Cohorts. Orphanet J. Rare Dis..

[4] Ferreira C.R. (2019). The Burden of Rare Diseases. Am. J. Med. Genet. Part A.

[5] *Code de La Santé Publique—Article L6316-1*; Volume L6316-1. https://www.legifrance.gouv.fr/codes/article_lc/LEGIARTI000038887059.

[6] *Décret N° 2010-1229 Du 19 Octobre 2010 Relatif à La Télémédecine*. 2010. https://www.legifrance.gouv.fr/loda/id/JORFTEXT000022932449.

[7] (2020). L’essor de La Télémédecine, Une Bascule Soudaine Rendue Possible Par Un Investissement Préalable Sur La Durée. In *Améliorer la Qualité du Système de Santé et Maîtriser les Dépenses—Propositions de L’Assurance Maladie Pour 2021*. https://assurance-maladie.ameli.fr/sites/default/files/2020-07_rapport-propositions-pour-2021_assurance-maladie_1.pdf.

[8] *Décret N° 2021-707 Du 3 Juin 2021 Relatif à La Télésanté*. 2021. https://www.legifrance.gouv.fr/jorf/id/JORFTEXT000043596730.

[9] Arrêté du 22 Septembre 2021 Portant Approbation de L’avenant n° 9 à la Convention Nationale Organisant les Rapports Entre les Médecins Libéraux et L’assurance Maladie Signée le 25 août 2016.

[10] Walkowiak D., Domaradzki J. (2020). Needs Assessment Study of Rare Diseases Education for Nurses and Nursing Students in Poland. Orphanet J. Rare Dis..

[11] Jahanshahi R., Nasirzadeh A., Farzan M., Domaradzki J., Jouybari L., Sanagoo A., Farzan M., Aghazadeh-Habashi K., Fallah Faraghe A., Bagheri S. (2022). Iranian Future Healthcare Professionals’ Knowledge and Opinions about Rare Diseases: Cross-Sectional Study. Orphanet J. Rare Dis..

[12] Sanges S., Farhat M.-M., Assaraf M., Galland J., Rivière E., Roubille C., Lambert M., Yelnik C., Maillard H., Sobanski V. (2020). Raising Rare Disease Awareness Using Red Flags, Role Play Simulation and Patient Educators: Results of a Novel Educational Workshop on Raynaud Phenomenon and Systemic Sclerosis. Orphanet J. Rare Dis..

[13] Soussand L., Kuchenbuch M., Messiaen C., Sandrin A., Jannot A.-S., Nabbout R. (2022). Impact of the COVID-19 Pandemic on the Care of Rare and Undiagnosed Diseases Patients in France: A Longitudinal Population-Based Study. Orphanet J. Rare Dis..

[14] Resseguier A.S., Gerbaud L., De Ruffray P., Vaillant-Roussel H., Pereira B., Ruivard M. (2017). Évaluation d’une plateforme de télé-expertise spécifique à la médecine interne. Rev. Med. Interne.

[15] Coppo P., Corre E., Rondeau E., Benhamou Y., Bachet A., Stépanian A., Veyradier A., Centre de référence des Microangiopathies Thrombotiques (2016). [Telemedicine in thrombotic microangiopathies: A way forward in rare diseases requiring emergency care]. Rev. Med. Interne.

[16] Devadula S., Langbecker D., Vecchio P., Tesiram J., Meiklejohn J., Benham H. (2020). Tele-Rheumatology to Regional Hospital Outpatient Clinics: Patient Perspectives on a New Model of Care. Telemed. J. E-Health.

[17] Piga M., Cangemi I., Mathieu A., Cauli A. (2017). Telemedicine for Patients with Rheumatic Diseases: Systematic Review and Proposal for Research Agenda. Semin. Arthritis Rheum..

[18] McDougall J.A., Ferucci E.D., Glover J., Fraenkel L. (2017). Telerheumatology: A Systematic Review. Arthritis Care Res..

[19] Gkrouzman E., Wu D.D., Jethwa H., Abraham S. (2020). Telemedicine in Rheumatology at the Advent of the COVID-19 Pandemic. HSS J..

[20] Cavagna L., Zanframundo G., Codullo V., Pisu M.G., Caporali R., Montecucco C. (2021). Telemedicine in Rheumatology: A Reliable Approach beyond the Pandemic. Rheumatology.

[21] Lampe C., Dionisi-Vici C., Bellettato C.M., Paneghetti L., van Lingen C., Bond S., Brown C., Finglas A., Francisco R., Sestini S. (2020). The Impact of COVID-19 on Rare Metabolic Patients and Healthcare Providers: Results from Two MetabERN Surveys. Orphanet J. Rare Dis..

[22] Clark J.M., Heifetz L.J., Palmer D., Brown L.M., Cooke D.T., David E.A. (2016). Telehealth Allows for Clinical Trial Participation and Multimodality Therapy in a Rural Patient with Stage 4 Non-Small Cell Lung Cancer. Cancer Treat. Res. Commun..

[23] Sabesan S., Zalcberg J. (2018). Telehealth Models Could Be Extended to Conducting Clinical Trials-a Teletrial Approach. Eur. J. Cancer Care.

[24] Doolittle G.C., Caracione A., Coulter J., Olson K., Knoebber-Carr K. (2018). Using Telemedicine to Increase Access to Cancer Clinical Trials for Patients in Rural Areas: A Feasibility Study. J. Clin. Oncol..

[25] Laggis C.W., Williams V.L., Yang X., Kovarik C.L. (2019). Research Techniques Made Simple: Teledermatology in Clinical Trials. J. Investig. Dermatol..

[26] Lee J.J., Burbury K., Underhill C., Harris S., Shackleton K., McBurnie J., McPhee N., Osmond F., Wilkins K., Baden P. (2020). Exploring Australian Regional Cancer Patients’ Experiences of Clinical Trial Participation via Telemedicine Technology. J. Telemed. Telecare.

[27] Gonçalves B.T., Baiocchi G. (2020). Telemedicine and Cancer Research during the COVID-19 Pandemic. J. Surg. Oncol..

[28] Takeda C., Guyonnet S., Ousset P.J., Soto M., Vellas B. (2020). Toulouse Alzheimer’s Clinical Research Center Recovery after the COVID-19 Crisis: Telemedicine an Innovative Solution for Clinical Research during the Coronavirus Pandemic. J. Prev. Alzheimers Dis..

[29] Izmailova E.S., Ellis R., Benko C. (2020). Remote Monitoring in Clinical Trials During the COVID-19 Pandemic. Clin. Transl. Sci..

