# Supplementary material for: Activities of Clinical Expertise and Research in a Rare Disease Referral Centre: A Place for Telemedicine beyond the COVID-19 Pandemic?
Source: Healthcare (Basel). 2023 Aug 31;11(17):2447. doi: 10.3390/healthcare11172447 (PMC10487162; doi:10.3390/healthcare11172447)
Supplement: Supplementary file 1 [file healthcare-11-02447-s001.zip › Tableau Supp 1.pdf]

Supplementary Table 1. Census form for specialist opinions given by physicians of the Internal Medicine - Clinical Immunology Department, CHU Lille, France

**1. Date of the opinion**<br>dd/mm/yyyy
**2. Context of the request for an opinion**<br>❍ Through our dedicated hotline (on-call physician)<br>❍ Outside our dedicated hotline
**3. Method used to contact you**<br>❍ Phone ❍ e-mail ❍ Other: ____________
**4. Person requesting the opinion**<br>❍ Hospital physician: Lille University Hospital<br>❍ Hospital physician: outside Lille University Hospital<br>❍ Private practitioner: generalist<br>❍ Private practitioner: specialist<br>❍ Patient (or relative)<br>❍ Other: ____________
**5. If the person requesting the opinion is a physician, indicate the speciality:**<br>Free text: ____________
**6. If the person requesting the opinion is a physician, indicate his/her workplace:**<br>Free text: ____________
**7. Patient who is the subject of the opinion**<br>❍ Patient already known to the Department ❍ Patient not known to the Department
**8. Reason the opinion is being requested**<br>Free text: ____________
**9. Bedside visit**<br>❍ Yes ❍ No
**10. Conclusion of the opinion**<br>❍ Admit patient in hospitalisation unit<br>❍ Schedule day-patient clinic visit<br>❍ Scheduled consultation<br>❍ Continuation of remote patient management (by phone or email)<br>❍ Ad hoc opinion<br>❍ Other: ____________
**11. Total time devoted to the opinion**<br>*If a visit outside the Department was needed, also include the duration of the visit*<br>hh:mm:ss
