# Supplementary material for: Activities of Clinical Expertise and Research in a Rare Disease Referral Centre: A Place for Telemedicine beyond the COVID-19 Pandemic?
Source: Healthcare (Basel). 2023 Aug 31;11(17):2447. doi: 10.3390/healthcare11172447 (PMC10487162; doi:10.3390/healthcare11172447)
Supplement: Supplementary file 1 [file healthcare-11-02447-s001.zip › Tableau Supp 2.pdf]

Supplementary Table 2. Feedback questionnaire on specialist opinions given by physicians of the Internal Medicine and Clinical Immunology Department, CHU Lille, France.

1. During the period covered by the census of medical opinions given in the Department, how exhaustive do you think you were in declaring your opinions?

	1	2	3	4	
Not at all exhaustive	❍	❍	❍	❍	Entirely exhaustive

2. On average, how many opinions would you say you forgot to report in a standard week (i.e. not on call)?

❍ < 2/week

❍ 2-5/ week

❍ 6-10/ week

❍ >10/ week

3. On average, how many opinions would you say you forgot to report during a week on call?

❍ < 2/ week

❍ 2-5/ week

❍ 6-10/ week

❍ >10/ week

❍ I was not on call during the period of the study

4. How exhaustive do you think you were in declaring your opinions given by phone?

	1	2	3	4	
Not at all exhaustive	❍	❍	❍	❍	Entirely exhaustive

5. How exhaustive do you think you were in declaring your opinions given by e-mail?

	1	2	3	4	
Not at all exhaustive	❍	❍	❍	❍	Entirely exhaustive

6. How exhaustive do you think you were in declaring your opinions given by post?

	1	2	3	4	
Not at all exhaustive	❍	❍	❍	❍	Entirely exhaustive

7. How exhaustive do you think you were in declaring your opinions that required a bedside visit?

	1	2	3	4	
Not at all exhaustive	❍	❍	❍	❍	Entirely exhaustive

8. How exhaustive do you think you were in declaring your opinions given to CHU Lille physicians?

	1	2	3	4	

Not at all exhaustive	❍	❍	❍	❍	Entirely exhaustive

9. How exhaustive do you think you were in declaring your opinions given to non-CHU Lille physicians?

	1	2	3	4	
Not at all exhaustive	❍	❍	❍	❍	Entirely exhaustive

10. How exhaustive do you think you were declaring your opinions given to generalist private practitioners?

	1	2	3	4	
Not at all exhaustive	❍	❍	❍	❍	Entirely exhaustive

11. How exhaustive do you think you were in declaring your opinions given to specialist private practitioners?

	1	2	3	4	
Not at all exhaustive	❍	❍	❍	❍	Entirely exhaustive

12. How exhaustive do you think you were in declaring your opinions given to patients (or a family member)?

	1	2	3	4	
Not at all exhaustive	❍	❍	❍	❍	Entirely exhaustive

13. How precise do you think you were in the declared duration of your opinions?

	1	2	3	4	
Not at all precise	❍	❍	❍	❍	Extremely precise

14. In general, how time-consuming did you find these declarations of opinions?

	1	2	3	4	
Not at all time-consuming	❍	❍	❍	❍	Extremely time-consuming

15. In general, how tedious did you find these declarations of opinions?

	1	2	3	4	
Not at all tedious	❍	❍	❍	❍	Extremely tedious

16. In general, were you able to declare these opinions at the time you gave them?

	1	2	3	4	
Not at all	❍	❍	❍	❍	Yes, at the same time
