# Supplementary material for: Activities of Clinical Expertise and Research in a Rare Disease Referral Centre: A Place for Telemedicine beyond the COVID-19 Pandemic?
Source: Healthcare (Basel). 2023 Aug 31;11(17):2447. doi: 10.3390/healthcare11172447 (PMC10487162; doi:10.3390/healthcare11172447)
Supplement: Supplementary file 1 [file healthcare-11-02447-s001.zip › Tableau Supp 3.pdf]

Supplementary Table 3. Questionnaire on views about telemedicine and clinical research for physician-investigators at the Internal Medicine and Clinical Immunology Department, CHU Lille, France

1. Have you heard of telemedicine?

❍ Yes ❍ No

2. Have you been an investigator in a clinical research study?

❍ Yes ❍ No

3. Have you included patients in a clinical research protocol?

❍ Yes ❍ No

4. Have you conducted or participated in a patient visit in the context of a protocol?

❍ Yes ❍ No

5. Do you think that having to visit the hospital more regularly would make patients more reluctant to take part in a clinical research protocol?

	1	2	3	4	5	
Strongly disagree	❍	❍	❍	❍	❍	Strongly agree

6. Do you think that a reduction in the number of on-site visits through the use of a telemedicine tool could increase the number of inclusions in a protocol?

	1	2	3	4	5	
Strongly disagree	❍	❍	❍	❍	❍	Strongly agree

7. If a clinical research study included the possibility of conducting some visits where the patient would stay at home and the physician stay in his/her office:
Do you think this could be less time consuming for you?

	1	2	3	4	5	
Strongly disagree	❍	❍	❍	❍	❍	Strongly agree

8. If a clinical research study included the possibility of conducting some visits where the patient would stay at home and the physician stay in his/her office:
Do you think this could be more convenient for the patient?

	1	2	3	4	5	
Strongly disagree	❍	❍	❍	❍	❍	Strongly agree

9. Do you think it could be dangerous for a patient included in a clinical research protocol to make some visits by teleconsultation?

	1	2	3	4	5	
Strongly disagree	❍	❍	❍	❍	❍	Strongly agree

10. Do you think telemedicine could improve patient management in clinical research?

	1	2	3	4	5	
Strongly disagree	❍	❍	❍	❍	❍	Strongly agree
