# Supplementary material for: Activities of Clinical Expertise and Research in a Rare Disease Referral Centre: A Place for Telemedicine beyond the COVID-19 Pandemic?
Source: Healthcare (Basel). 2023 Aug 31;11(17):2447. doi: 10.3390/healthcare11172447 (PMC10487162; doi:10.3390/healthcare11172447)
Supplement: Supplementary file 1 [file healthcare-11-02447-s001.zip › Tableau Supp 4.pdf]

Supplementary Table 4. Questionnaire on views about telemedicine and clinical research for patients included in a clinical research protocol at the Internal Medicine and Clinical Immunology Department, CHU Lille, France

You have agreed to take part in a clinical research protocol.
**1. Do you have to visit the hospital more frequently since the start of the study?**<br>❍ Yes ❍ No
**2. Do you find it difficult to organise your visits to the hospital?**<br>❍ Yes ❍ No<br>If Yes, why?: ____________________
**3. Have you already heard of telemedicine and teleconsultation?**<br>*Definition of telemedicine: telemedicine is a form of medical practice conducted remotely using information and communication technologies to establish a link between patients and their doctors or between health professionals.*<br>*Definition of teleconsultation: remote consultation between a patient (whether or not accompanied by a health professional) and a doctor.*<br>❍ Yes ❍ No
**4. If you could choose between a consultation at the hospital and a teleconsultation at home, which would you choose?**<br>❍ Hospital ❍ Home
**5. Would you feel reassured if, in the context of the research protocol, there were additional follow-up visits by teleconsultation?**

	1	2	3	4	5	
Not at all reassured	❍	❍	❍	❍	❍	Very reassured

**6. Do you think telemedicine could improve the care you receive?**<br>❍ Yes ❍ No
**7. If a clinical research study included the possibility of conducting some visits where the patient would stay at home and the physician stay in his/her office using a telemedicine tool:**<br>**Do you think that would take you less time?**<br>❍ Yes ❍ No
**8. If a clinical research study included the possibility of conducting some visits where the patient would stay at home and the physician stay in his/her office using a telemedicine tool:**<br>**Do you think it would be more convenient for you?**<br>❍ Yes ❍ No
**9. If a clinical research study included the possibility of conducting some visits where the**

patient would stay at home and the physician stay in his/her office using a telemedicine tool:

Do you think that could be dangerous for you?

	1	2	3	4	5	
Do not agree at all	❍	❍	❍	❍	❍	Agree entirely
